# Investigating the Effects of the Physicochemical Properties of Cellulose-Derived Biocarbon on Direct Carbon Solid Oxide Fuel Cell Performance

**DOI:** 10.3390/ma17143503

**Published:** 2024-07-15

**Authors:** Bartosz Adamczyk, Magdalena Dudek, Anita Zych, Marcin Gajek, Maciej Sitarz, Magdalena Ziąbka, Piotr Dudek, Przemysław Grzywacz, Małgorzata Witkowska, Joanna Kowalska, Krzysztof Mech, Krystian Sokołowski

**Affiliations:** 1 Faculty of Energy and Fuels, AGH University of Krakow, Mickiewicza 30 Av., 30-059 Krakow, Poland; zycha@agh.edu.pl (A.Z.); grzywacz@agh.edu.pl (P.G.); 2 Faculty of Materials Science and Ceramics, AGH University of Krakow, Mickiewicza 30 Av., 30-059 Krakow, Poland; mgajek@agh.edu.pl (M.G.); msitarz@agh.edu.pl (M.S.); ziabka@agh.edu.pl (M.Z.); 3 Faculty of Mechanical Engineering and Robotics, AGH University of Krakow, Mickiewicza 30 Av., 30-059 Krakow, Poland; pdudek@agh.edu.pl; 4 Faculty of Metals Engineering and Industrial Computer Science, AGH University of Krakow, Mickiewicza 30 Av., 30-059 Krakow, Poland; witkowsk@agh.edu.pl (M.W.); joannak@agh.edu.pl (J.K.); 5 Academic Centre for Materials and Nanotechnology, AGH University of Krakow, al. A. Mickiewicza 30, 30-059 Krakow, Poland; kmech@agh.edu.pl (K.M.); krysok@agh.edu.pl (K.S.)

**Keywords:** biochar, cellulose, circular economy, direct carbon solid oxide fuel cells, power sources, wastes

## Abstract

This paper presents a study of the characteristic effects of the physicochemical properties of microcrystalline cellulose and a series of biocarbon samples produced from this raw material through thermal conversion at temperatures ranging from 200 °C to 850 °C. Structural studies revealed that the biocarbon samples produced from cellulose had a relatively low degree of graphitization of the carbon and an isometric shape of the carbon particles. Based on thermal investigations using the differential thermal analysis/differential scanning calorimeter method, obtaining fully formed biocarbon samples from cellulose feedstock was possible at about 400 °C. The highest direct carbon solid oxide fuel cell (DC-SOFC) performance was found for biochar samples obtained via thermal treatment at 400–600 °C. The pyrolytic gases from cellulose decomposition had a considerable impact on the achieved current density and power density of the DC-SOFCs supplied by pure cellulose samples or biochars derived from cellulose feedstock at a lower temperature range of 200–400 °C. For the DC-SOFCs supplied by biochars synthesised at higher temperatures of 600–850 °C, the “shuttle delivery mechanism” had a substantial effect. The impact of the carbon oxide concentration in the anode or carbon bed was important for the performance of the DC-SOFCs. Carbon oxide oxidised at the anode to form carbon dioxide, which interacted with the carbon bed to form more carbon oxide. The application of biochar obtained from cellulose alone without an additional catalyst led to moderate electrochemical power output from the DC-SOFCs. The results show that catalysts for the reverse Boudouard reactions occurring in a biocarbon bed are critical to ensuring high performance and stable operation under electrical load, which is crucial for DC-SOFC development.

## 1. Introduction

Direct carbon solid oxide fuel cells (DC-SOFCs) are a promising subtype of direct carbon fuel cells (DCFCs). The latter converts chemical energy from carbonaceous fuels directly into electricity. The products of the electrochemical oxidation of carbon in DCFCs are waste heat and carbon dioxide (CO_2_). A high concentration of CO_2_ in the outlet gas stream from the fuel cells’ anode chamber facilitates capture and further utilisation [1,2]. In the classic concept of a DC-SOFC, carbon can be electrochemically oxidised directly into CO_2_ via the reaction C + O^2−^→CO_2_ + 4e^−^. It can also be formed in a sequence of electrochemical reactions as follows: C + O^2−^→CO + 2e^−^ and CO + O^2−^→CO_2_ + 2e^−^. DC-SOFCs supplied by pulverised carbonaceous solid fuels operate at temperatures of 700–850 °C. Under these conditions, the Boudouard reaction (1) should be considered—that is, C + CO_2_→CO. Reaction (1) involves CO_2_ and carbon as reactants and can serve as an additional source of carbon oxide (CO), which is consumed during reaction CO + O^2−^→CO_2_ + 2e^−^ [3,4]. Various solid carbon fuels have been investigated for DC-SOFC operation. The power density (P) obtained from DC-SOFCs ranges from ~40 mW/cm^2^ to ~300 mW/cm^2^. It is well-known that disordered carbon particles (characterised by a low graphitization level) in solid carbon fuels exhibit better chemical activity in both the Boudouard reaction and the electrochemical anode oxidation process [5,6,7,8]. The strategic goal in developing DCFCs is to find cheap and readily available solid fuel. The most frequently proposed among these are fossil fuels and biomass. Pulverised hard coal, lignite, and coke are also considered possible solid fuels for DC-SOFCs. The values achieved for the output power (Pmax) of a DC-SOFC supplied with powdered coal or lignite are above 100 mW/cm^2^ [9,10,11,12,13].

The valorisation of renewable-waste biomass resources into value-adding biofuels has attracted worldwide interest. Recent efforts have focused on searching for renewable carbon derived from different sources of waste biomass. As a fuel, biochar produced from waste materials offers many advantages. It is inexpensive and easy to store, and no particular logistics are required for its transportation [14,15]. Unlike fossil fuels, biocarbons contain no mercury, almost no sulphur, and little nitrogen (N_2_), producing very little ash. Accordingly, DC-SOFCs may be installed where local waste biomass is used. Since DC-SOFCs operate at high temperatures of 700–850 °C, they must be started at room temperature and then gradually heated until they reach the target operating temperature of 850 °C. Under these conditions, solid fuels in DC-SOFCs can undergo thermochemical conversion, releasing gases, liquids, and solids during decomposition. In turn, the denoted medium can serve as fuel to supply the DC-SOFCs [16,17,18].

Biomass with different chemical compositions and proportions of lignin, cellulose, and hemicellulose comprise a wide range of plants that, in addition to their nutritional properties as food, can also serve as biofuel energy sources [19,20,21]. Depending on the type of biomass, these components are present in different ratios. In addition to the ingredients listed above, water, minerals, lipids, and proteins are always present. Mineral substances are an integral part of plant biomass. Individual types of biomass differ in their total mineral content and qualitative and quantitative chemical compositions. Some mineral matter components are valuable natural catalysts [22,23,24,25].

To date, no systematic studies exist on the quantitative effects of individual components (e.g., cellulose and hemicellulose) on electrochemical carbon oxidation in SOFCs. Despite intensive research, optimised sources of biomass fuels from particular groups have not yet been found.

The goal of this paper is to determine the physiochemical properties of biochar powders obtained from cellulose feedstock and their impact on direct carbon solid oxide fuel cell (DC-SOFC) performance. The special input given is the investigation of the experimental factors that influence the mechanism and kinetics of the gasification process that can occur in DC-SOFCs. The second part of the manuscript is dedicated to analysing the process of electrochemical oxidation of carbon fuel in anodes in DC-SOFCs. In these investigations, the analysis of the contribution of the solid form of carbon fuel (i.e., carbon particles) as well as CO as gaseous fuel for DC-SOFC performance has been provided. The additional effect of pyrolytic products involving different organic carbon compounds, which evolve under the decomposition of cellulose during pyrolysis, has also been considered for the current density and electrical power of such fuel cells. According to the authors’ knowledge, these factors are strategic goals for the optimisation of biochar properties for utilisation in DC-SOFCs.

## 2. Materials and Methods

### 2.1. Description of the Preparation of Biochar Samples by Thermal Processing Cellulose Raw Materials in an Inert Gas Atmosphere

Commercially available microcrystalline cellulose (Merck, Darmstadt, Germany) was chosen as the source material. Cellulose feedstock was initially sifted through sieves of varying sizes. Then, samples of much smaller weight (approximately 10 g) were used to prepare the solid carbon fuels. These smaller portions were subjected to thermal conversion at a temperature range of 200–850 °C. Torrefied biomasses or chars were obtained by thermally treating the cellulose powders in a quartz reactor at 200–850 °C in an N_2_ gas atmosphere for 1 h. The flow of N_2_ was established at 50 mL/min.

**Figure 1:**
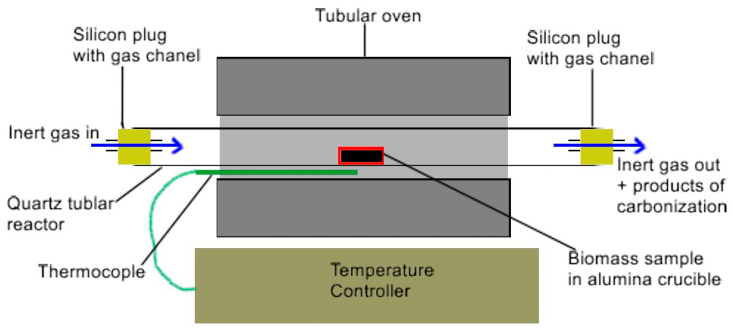
Scheme of the reactor for the thermal processing of biomass samples.

Figure 1 shows the experimental setup for biocarbon preparation via the pyrolysis method. This experimental reactor was built by the authors of this work in accordance with the developed project. The main advantage of this flow reactor is the possibility of producing biocarbon from different organic waste products from biomass under similar conditions as with the DC-SOFC setup. They are then used as fuels in solid oxide fuel cells (DC-SOFCs). The experimental setup followed, as described in [26].

The cellulose-derived solid carbon fuels were passed through a 0.02 mm mesh steel sieve (Conbest, Kraków, Poland). The resulting powdered samples were the subject of further analytical and electrochemical investigations.

The following abbreviations are used throughout the text for the tested biomass fuel samples. The entire sample series is represented by CMK, which refers to the original chemical compound, cellulose microcrystalline. The numbers 200, 300, 400, 600, and 850 represent the temperatures of the thermal treatment of the raw cellulose, while 0 refers to the original sample that did not undergo any thermal treatment. To obtain the same conditions, the temperature was increased by 5 °C/min until it reached the desired temperature. In this way, a series of five biocarbon samples were obtained. Details of the sample synthesis are presented in Table 1.

The original raw samples, as well as the thermally treated samples, were subjected to further physicochemical investigations and were also investigated as solid fuels in SOFCs. Once removed from the reactor, the biochar samples were homogenised and crushed in an agate mortar.

### 2.2. Chemical and Structure Analyses of All Samples

The raw microcrystalline cellulose (CMK-0) and all biocarbon powders were subjected to elemental analysis. The primary elements (carbon, hydrogen [H_2_], and sulphur) were determined using the ELTRA CHS-580 chemical analyser (ELTRA GmbH, Haan, Germany). The X-ray diffraction (XRD) method was used to evaluate the phase composition of the raw cellulose powder and the synthesised biocarbon samples. Diffraction analysis was conducted on a Siemens D500 diffractometer using a filtered lamp with a copper anode (λ = 0.15418 nm) and the step count method (∆2θ = 0.04°, counting time τ = 10 s/step, angle range 2θ = 10°–120°). The XRD patterns recorded for the biocarbon derived from cellulose enabled inspection of the phase composition. The residual cellulose content in the biocarbon was monitored, particularly for the low-temperature synthesised samples. In the case of the monophase biocarbon samples, the degree of graphitization versus the applied temperature of the thermal conversion of the samples was observed.

The XRD method was also applied to determine the phase composition of the materials used as components for constructing DC-SOFCs, which were supplied by solid fuels derived from cellulose as a feedstock. The carbon particles’ Raman spectra were recorded using confocal Raman spectrometer Lab-RAM HR 800 (HORIBA, Longjumeau, France SAS) equipped with 532 nm laser.Mid-infrared spectroscopy (MIR) studies of the chemical functional groups in the biochars were performed using a Bruker Vertex 70v spectrometer (Bruker, Billerica, MA, USA). The standard KBr palette method was used, and 128 scans were accumulated with a resolution of 4 cm^−1^ in the range of 4000–400 cm^−1^ to decipher some chemical groups in the biochars.

### 2.3. Scanning Electron Microscopy Investigations of the Obtained Samples

The morphology and chemical composition of the CMK-0 samples and the prepared carbon samples derived from the cellulose after thermal treatment were observed using scanning electron microscopy (SEM) with an Apreo 2S Low vac SEM, Thermo Fisher Scientific (Waltham, MA, USA) with APEX^TM^ software (https://www.edax.com/products/eds/apex-software-for-eds, accessed on 15 June 2024) for energy-dispersive X-ray spectroscopy (EDX, EDAX) (Eindhoven, The Netherlands). The observations were carried out in high vacuum conditions using the ETD detector. The biochar particles’ morphological structure and their particle size distribution were noted. The chemical composition of the carbon particles was monitored. The SEM method was also used to observe the microstructure of the electrodes and the electrolyte-based materials. These were used as components for the investigated solid oxide fuels supplied through the biocarbon-based powders.

### 2.4. X-ray Photoelectron Spectroscopy Analyses of Biochar Derived from Cellulose

X-ray photoelectron spectroscopy (XPS) analyses were carried out in a PHI Versa Probe II Scanning XPS system (Lake Drive East, Chanhassen, MN, USA) using monochromatic Al Kα (1486.6 eV) X-rays that were focused on a 100 µm spot and scanned over an area of 400 µm × 400 µm. The photoelectron take-off angle was 45°, and the pass energy in the analyser was set to 117.50 eV (0.5 eV step) for the survey scans and 46.95 eV (0.1 eV step) to obtain high-resolution spectra of C 1s, O 1s, and Si 2p. A dual beam charge compensation with 7-eV Ar^+^ ions and 1-eV electrons was used to maintain a constant sample surface potential, regardless of the sample conductivity. All XPS spectra were charge-referenced to the unfunctionalised saturated carbon (C–C) C 1s peak at 285.0 eV. The operating pressure in the analytical chamber was less than 3 × 10^−9^ mbar. The deconvolution of the spectra was carried out using PHI MultiPak software (v. 9.9.3). The spectrum background was subtracted using the Shirley method [27].

### 2.5. Investigations Using Thermal Analysis Methods Performed on the Biochar Samples

The thermal effects that occurred during the heating of the solid carbon fuel in the temperature range of 25–1000 °C in the N_2_ gas stream were measured using the differential thermal analysis (DTA) and thermogravimetric (TG) methods (STA 449 F3Jupiter^®^ simultaneous thermal analyser, provided by Netzsch, Krakow, Poland The samples (approximately 50 mg) were ramped up at a 10 °C/min rate in a platinum crucible. The measurements were carried out in an N_2_ gas atmosphere.

The kinetics of the gasification of the charred biocarbon samples in the steam atmosphere were studied using the thermovolumetric method. The measurements were conducted using unique laboratory equipment to investigate gasification kinetics under isothermal conditions in a broad water (H_2_O) pressure range. This method was previously used to study biochar obtained from pistachio shells.

The efficiency of the chemical reactivity of the solid carbon particles under the gasification process can be expressed as:

dV/dt = f(t)(1)

where dVi/dt equals the rate of formation of a given product in cm^3^/min∙g, and t equals time in minutes.

These dependencies (dV/dt) were determined by the measurements of the concentration of gaseous products in the post-reaction gas, the flow of which was maintained at a constant level during the entire measurement.

The release rate of a given product over time can be calculated by:

(dVi)/dt = V ci(t)(2)

where V equals the volume flow of the reaction gas in cm3/min, and ci(t) equals the concentration of a given product in the post-reaction gas at time (t) in the percentage of volume. The apparatus and the study of coal and biomass gasification are described in [28,29].

### 2.6. Analysis of Carbon Oxide Production during the Thermal Processes Occurring in the Carbon Bed Formed in a Direct Carbon Solid Oxide Fuel Cell Anode Chamber

Biocarbon samples as solid fuels were introduced to an anode chamber of the DC-SOFC. A complete DC-SOFC was placed in an electric tube furnace. The experimental setup, designed to investigate solid-button oxide fuel cells, was heated to 850 °C. The temperature increase ramp was 5 °C/min. N_2_ or CO_2_ gas was introduced into the DC-SOFC anode fuel chamber. The flow rate of the gas was established at 20 cm^3^/min. Gas sampling for chemical analyses was performed approximately 30 min after stabilising the temperature. The chemical composition of the exhaust gases from the DC-SOFC anode chamber at stabilised steady-state conditions was analysed in the temperature range of 700–850 °C, with increases in steps of 50 °C. The gas samples were taken from the anode chamber using a laboratory syringe with clamps attached. The gas samples (approximately 5 mL) were injected into a Sep-Pack^®^ N column using a Thermo Scientific 1310 chromatograph. provided by Alchem, Warsaw, Poland).

The chemical analysis of the gas mixtures aimed to obtain data on the variation in the CO/CO_2_ volume ratio in the anode chamber as a function of temperature. These tests reflected the chemical composition of the gases resulting from the interaction between the surrounding gas atmosphere and a fixed carbon bed formed in the DC-SOFC. The tests provided reference data for the experimental conditions under which the DC-SOFC operated at the applied electrical load. This method aimed to study the evolution of the gas from the biocarbon samples derived from cellulose, focusing on CO and CO_2_ (which formed in the anode chamber), as well as to consider their impact on possible Pmax variation in DC-SOFCs [26,30].

### 2.7. Analysis of the Electrochemical Performance of Direct Carbon Solid Oxide Fuel Cells Supplied by Solid Fuels Derived from Cellulose

The direct electrochemical oxidation of carbon was studied using four types of electrochemical cells:

(A) C|8YSZ|LSM-GDC|LSM|O_2_
(B) C|Ni-YSZ|YSZ|LSM-GDC|LSM|O_2_

In the case of DC-SOFC (A), designed to study the electrochemical process of carbon particles, the oxidation process of 8 mol% of Y_2_O_3_ in ZrO_2_ (8YSZ) was used in this study. The composite layered cathode La_0.8_Sr_0.2_MnO_3_ (LSM) and composite cathode material made of LSM + 10 mol % Gd_2_O_3_ in CeO_2_ (LSM-GDC) were applied to the investigated DC-SOFCs. Ni-YSZ is a cermet anode material composed of 50 vol% of 8YSZ and 50 vol% of Ni and is used as an anode in DCFCs. All electrochemical measurements were carried out within the 700–850 °C temperature range. N_2_ (purity 5N) was introduced as a shielding gas for the DC-SOFC (A) and (B) anodes. An electrochemical station with a PGSTAT 300 N potentiostat equipped with FRA was used in the investigations. The electrochemical DC-SOFC setup and measurements are described in our previously published papers [31,32].

## 3. Results and Discussion

The experimental results and their implications are discussed in this section.

### 3.1. Evaluation of Biochar Physicochemical Properties

One factor determining the achieved values of current density (I) and power (P) from DC-SOFCs is the content of basic elements in the organic material—that is, the content of ca. Table 2 shows the results from the analytical tests of the synthesised carbon materials obtained from cellulose.

As shown in Table 2, the carbon content of the biochar samples increased with the microcrystalline cellulose samples’ heating temperature. This phenomenon was caused by an increase in the samples’ degree of carbonisation at increasingly higher temperatures. A different trend was observed in the case of H_2_ as the biochar heating temperature increased; a decrease in H_2_ content was observed. The biochar samples obtained from cellulose had a very low sulphur content.

The results presented in Table 2 indicated that the obtained chars had a carbon content ranging from ~42 wt% to ~90 wt%. The obtained carbon content in the biochar samples was sufficient for use as solid fuels in DC-SOFCs. Based on the analysis of available experimental research results from DC-SOFCs supplied by solid carbonaceous fuels produced from different carbonaceous materials and the correlation between the total elemental carbon content in carbon char samples and changes in the I and P derived from DC-SOFCs, it follows that the best electrical parameters for DC-SOFCs were those characterised by a carbon content higher than ~60 wt% in the samples [33,34].

The obtained biocarbon-based samples (CMK-200 to CMK-850) from cellulose feedstock via thermal treatment were characterised by a lack of mineral matter. Moreover, this series of biocarbon samples enabled the examination of the electrochemical activity of carbon particles and the impact of individual gas components released during the gradual conversion of cellulose, which can take place in the anode chamber of DC-SOFCs.

**Figure 2:**
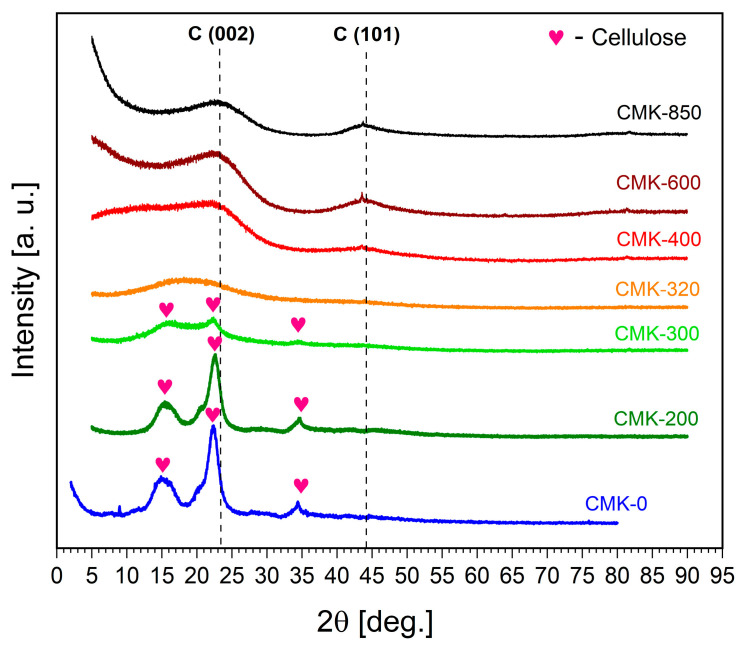
X-ray diffraction patterns recorded for CMK-0 and biochar obtained from cellulose via thermal treatment (samples CMK-200 to CMK-850).

Figure 2 shows the XRD patterns recorded for pure CMK-0 and the biochar samples (CMK-200 to CMK-850) obtained via thermal treatment in the temperature range of 200–850 °C.

Figure 2 also shows the recorded XRD patterns for biochar samples obtained from microcrystalline cellulose through additional heating in an oxygen-free atmosphere at temperatures ranging from 200 °C to 850 °C. In the CMK-200 sample, cellulose reflex patterns were visible. (110) in the ca. 15–16° position was observed as a broad peak but was composed of two peaks—that is, (200) in the 22.5° position and (004) in the 34° position. Based on this diffraction pattern recorded for the CMK-300 sample, it could be concluded that heating in these conditions led to the formation of two-phase samples composed of the carbon phase and cellulose residues. Increasing the sample heating temperature to 300 °C led to an increase in the degree of conversion of the cellulose phase to the amorphous carbon phase.

In the case of the partially carbonised CMK-300 sample, reflex patterns also remained, suggesting that a significant portion of cellulose had been decomposed, but only trace amounts were observed. In the CMK-320 sample, only the broad peak was visible in the 15–20° region, where the cellulose reflex pattern was present. In the CMK-400 biochar sample, residues of the cellulose pattern (except for elevated signal levels in the 5–25° region) were present.

Emerging weak peaks in the C(002) and C(001) regions were also visible, characteristic of graphitic structures. For the CMK-600 sample, no traces of reflex characteristics for biomass were observed, while the previously mentioned graphitic peaks had a better shape and intensity than the CMK-400 sample. Sample CMK-850 was similar to CMK-600, but the graphitic reflexes were slightly more mature. Based on the XRD tests, it was found that solid fuel samples heated at 400–850 °C were monophase carbon materials. These results showed close agreement with the data in [35].

**Figure 3:**
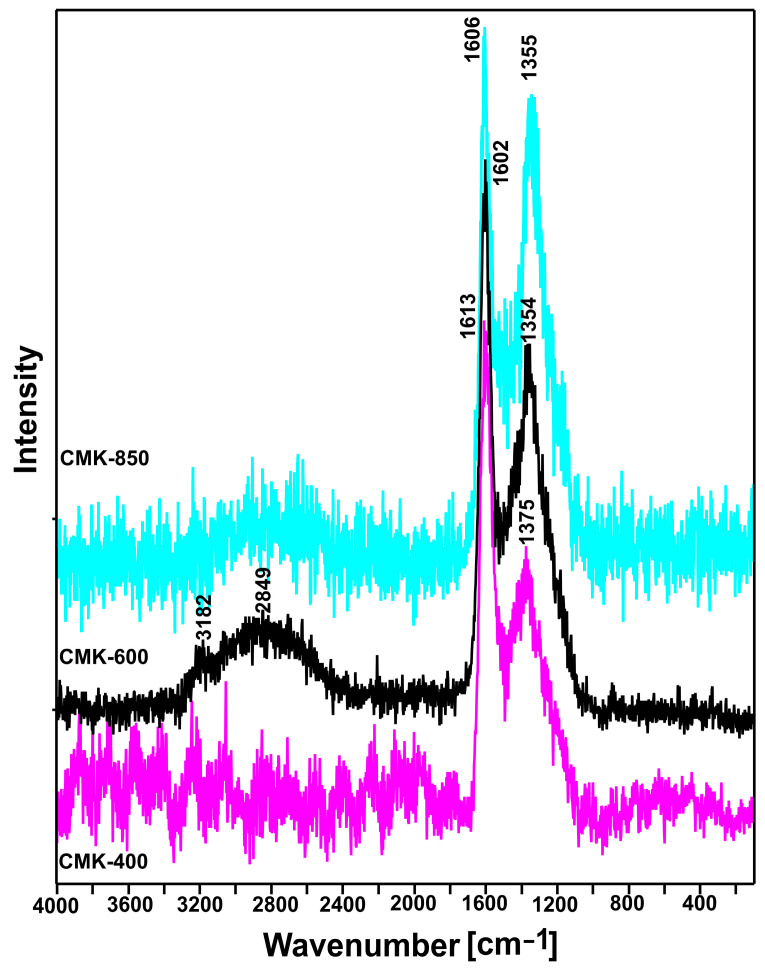
Raman spectra recorded for the CMK-400 to CMK-850 char samples.

The Raman spectra obtained for the microcrystalline cellulose samples that were heat-treated from 400 °C to 850 °C are presented in Figure 3.

For heat-treated microcrystalline cellulose, two bands with a large half-width were observed at approximately 1600 cm^−1^ (the so-called G band) and 1350 cm^−1^ (the so-called D band) [36]. Literature data indicate that the appearance of these two characteristic bands in the Raman spectrum indicates the presence of graphitic carbon. The G band is associated with stretching vibrations in the plane of carbon pairs in sp2 hybridization, while the D band is associated with symmetric breathing vibrations of hexagonal carbon rings. Based on the half-width of these bands and their intensity ratio (ID/IG), it is possible to conclude about the degree of crystallinity and defects of the graphite phase [37]. As can be seen in Figure 3, an increase in temperature leads to a slight decrease in the half-width of the bands, which indicates an increase in the degree of crystallinity. This conclusion is confirmed by the appearance of bands at approximately 3180 and 2850 cm^−1^, which are the D+D and D+G combination bands, respectively [38]. On the other hand, however, attention should be paid to the change in the intensity of the D and G bands. As the temperature increases, the intensity of the D band increases significantly, which indicates an increase in the degree of defects in the graphite phase.

**Figure 4:**
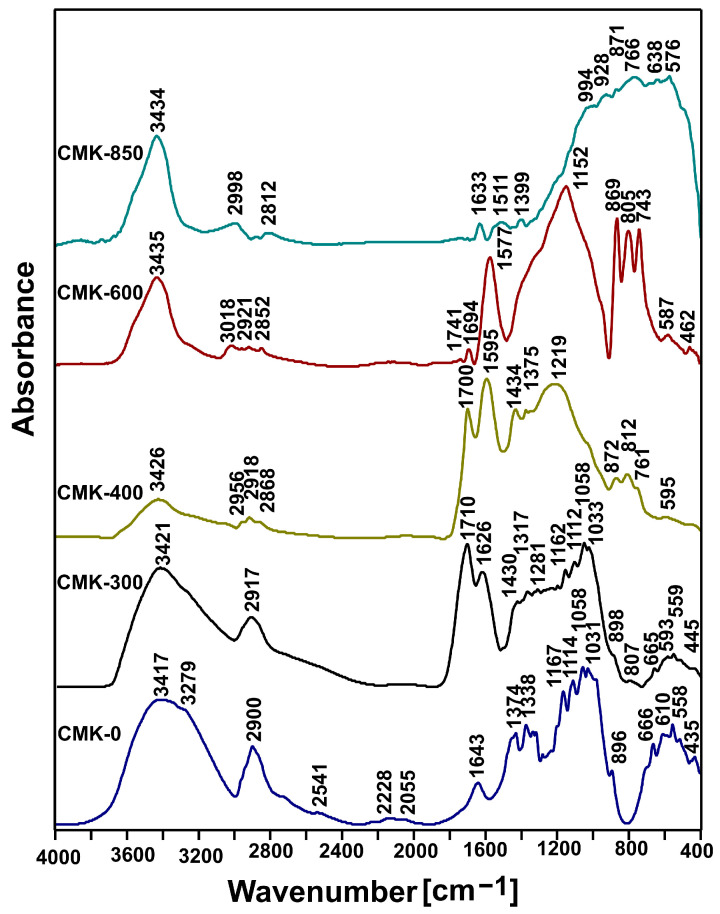
Mid-infrared spectroscopy spectra recorded for the original cellulose CMK-0 and the biochar samples obtained via thermal treatment.

Mid-infrared (MIR) spectroscopy was used to investigate the evolution of the chemical composition of organic compounds during the thermal conversion of cellulosic feedstock. Figure 4 shows the MIR spectra recorded for the starting sample (CMK-0) and biochar samples obtained in the temperature range 300–850 °C (CMK-300 to CMK-850). As the temperature increases to 400 °C, a sharp decrease in the intensity of the band at approximately 3420 and 1640 cm^−1^ associated with the vibrations of hydroxyl groups is visible, indicating the occurrence of dehydration or dehydrogenation. So, partial cellulose pyrolysis in the 300–400 °C temperature range produced torrefied samples, which characterised a reduction in intensity for the OH absorption group.

By contrast, a slight increase in the intensity of the presence of the OH group was observed for the well-carbonised samples, CMK-600 and CMK-850. The output spectrum (CMK-0) also shows other bands characteristic of cellulose, important from the point of view of describing the pyrolysis process–bands at: 2900 cm^−1^ (C-H), 1380–1330 cm^−1^ (C=C), 1170–1110 cm^−1^ (-CH_3_) 1030–1060 cm^−1^ (C-O-C). As indicated in the literature [39,40], in order to describe the course of the process, it is crucial to consider changes in the intensity ratio of the bands associated with C=O:C=C. As you can see, the C=O:C=C ratio decreased with the increase in temperature transformation (samples CMK-300 to CMK-400). This was due to an increase in C=O formation from hydrocarbon decomposition reactions such as dehydration and cracking. This was due to the increase in the formation of C=O from the reaction decompositions of the hydrocarbons, such as dehydration and cracking. A decrease in C=O was observed in samples CMK-600 and CMK-850, most likely because of the direct reaction of biochar pyrolysis. This led to a decrease in the C=O group at higher temperatures.

XPS methods were used to obtain additional information about the atomic composition and, particularly, the surface concentration of chemical bonds present on biochar sample CMK-850 after thermal treatment at 850 °C.

**Figure 5:**
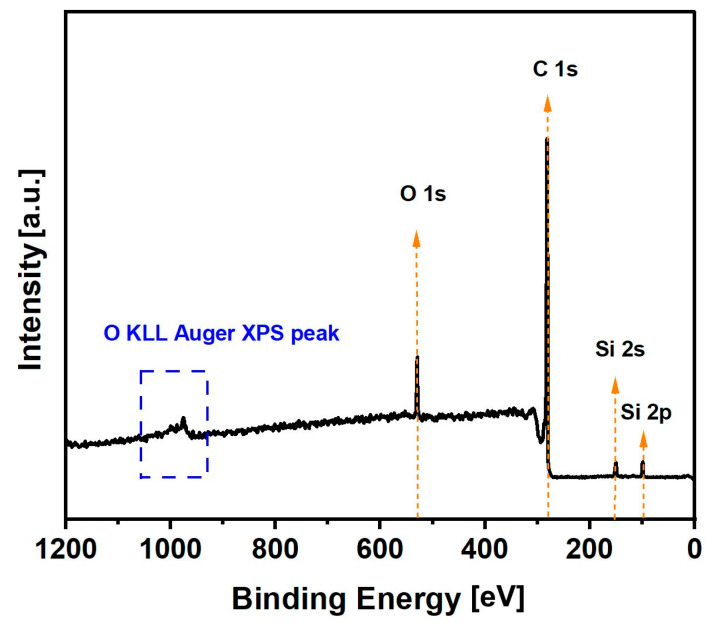
XPS survey spectrum for the carbonised sample CMK-850.

The chemical elements detected on this biocarbon were mainly carbon (C 1s line) and oxygen (O 1s line), with small traces of silicon contamination (Figure 5). Analysing the total content of individual elements, it was observed that the biochar samples obtained from the cellulose feedstock were characterised by high purity.

**Figure 6:**
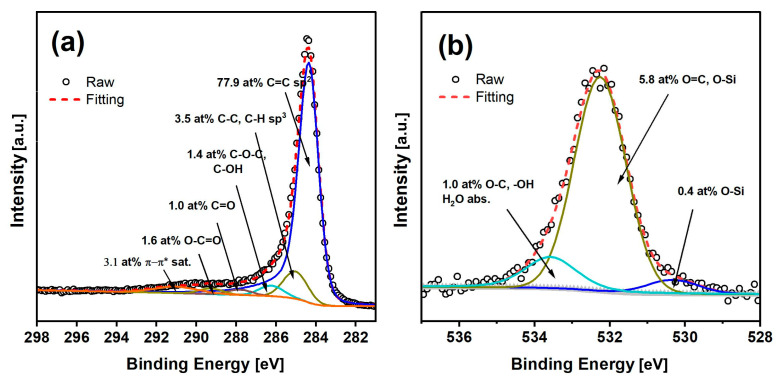
Deconvolution of C1 (**a**) and O1 (**b**) core excitations.

The collected high-resolution spectra for C 1s and O 1s are shown in Figure 6. The C 1s spectrum of the sample was fitted with six components. The first is most localised at a binding energy of 284.4 eV, indicating C=C (sp^2^) bonds. The maximum related to the second component is visible at 285.1 eV, indicating the presence of C-C (sp^3^) bonds. The maximum at 286.3 eV corresponding to line 3 shows the presence of C-O-C and/or C-OH groups. Line 4 of the maximum appears at 287.8 eV, indicating the presence of C=O groups. Line 5 has a peak localised at 287.8 eV, showing the presence of O-C-O bonds, while the last line of maximum at 291.0 eV is attributed to π->π* shake-ups. The shake-up excitation originates from the sp2 carbon and its aromatic forms and is an additional parameter confirming the presence of this type of bond. The O 1s spectrum was fitted with three lines, with the first line cantered at 530.7 eV, which indicates the presence of O-Si bonds; the second line of maximum at 532.4 eV coming from either O-Si and/or O=C type groups; and the last line of maximum found at 533.8 eV, which can originate from O-H and/or O-C type bonds and/or adsorbed H_2_O.

The Si 2p spectrum was fitted with two doublet structures (p3/2–p1/2 doublet sepa-ration equals 0.6 eV), with the main 2p3/2 line centred at 101.7 eV, which indicates the presence of C-Si-O type compounds like, e.g., silicone or siloxanes, and the second 2p3/2 line cantered at 103.1 eV, coming from silica species [41,42,43].

SEM was used to analyse the morphology of the carbon particles in all biochar samples of the CMK series obtained from raw cellulose. The morphological particularities of the biochar powders obtained from CMK-0 were an important factor in the investigation of the electrochemical oxidation processes of carbon fuels in DC-SOFC cells.

**Figure 7:**
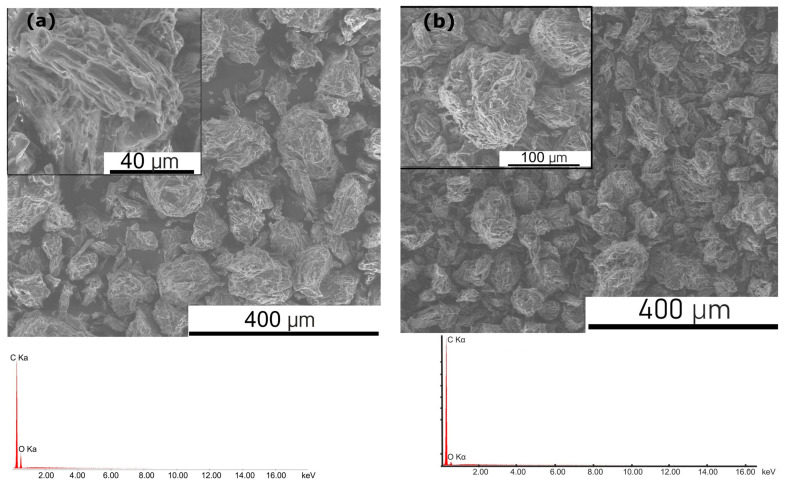

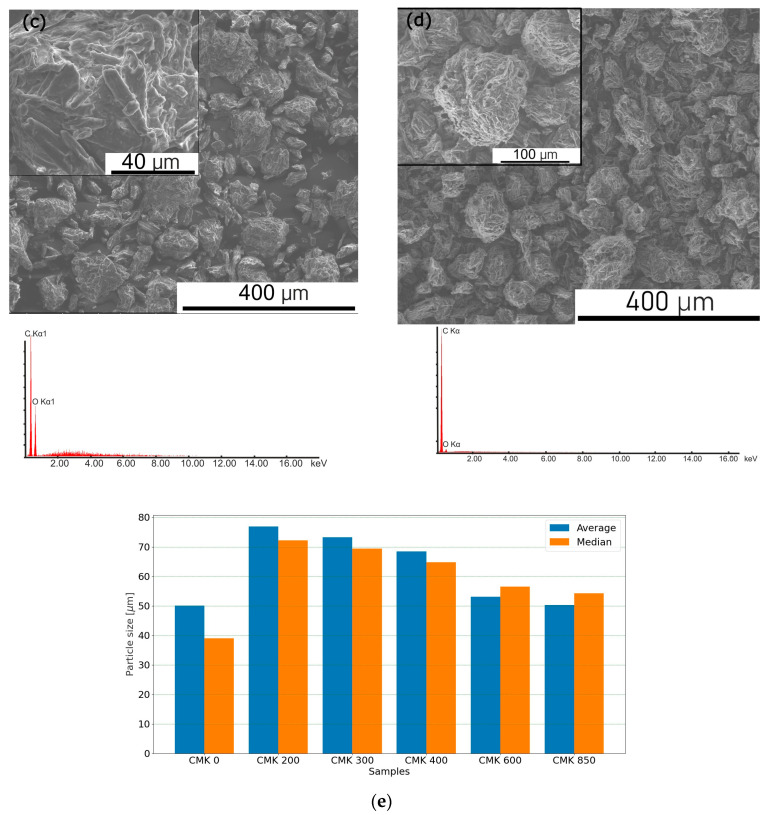
(**a**,**b**) Scanning electron microscopy images of the pure cellulose CMK-0 and biochar CMK-300 samples, including quality chemical analysis performed using the energy-dispersive X-ray spectroscopy method. (**c**,**d**) Scanning electron microscopy images of biochar CMK-600 and CMK-850, including quality chemical analysis performed using the energy-dispersive X-ray spectroscopy method. (**e**) The particle size distribution determined the raw cellulose CMK-0 and the carbonised samples from the CMK series.

Figure 7a,b provide SEM images of the CMK-0 and CMK-300 samples. They also show the EDX analysis of the carbon particles.

As seen in Figure 7a,b, in the case of the pure cellulose used as feedstock, the particles ranged from ~5 μm to 150 μm. For the partially carbonised sample CMK-300, solid carbon particles with regular and irregular shapes were observed. Their particle size varied from ~7 μm to ~240 μm. EDS analysis of the carbon particles’ chemical composition indicated that the CMK-300 sample was characterised by high purity. The measured signals from carbon or oxygen are marked on the spectrum.

In Figure 7c,d, the SEM images of CMK-600 and CMK-850 are presented, along with the chemical EDX analysis.

The recorded SEM images illustrated that with an increase in temperature for monophase biocarbon samples CMK-600 or CMK-850, the average particle size of carbon particles decreases with increasing temperature. Figure 7c provides the SEM image of the carbon particles. In the case of CMK-600, the particle size ranged from ~7 μm to ~140 μm. CMK-850, the biochar synthesised at the highest temperature, was characterised by a lower carbon size particle range from ~5 μm to ~111 μm. Figure 7e shows the collective results of the particle-size distribution analysis for the investigated sample family: CMK-0 as the pure cellulose sample, the two-phase torrefied samples CMK-200 and CMK-350, and the single-phase biochar samples CMK-400, CMK-600, and CMK-850.

It can be concluded from Figure 7e that in the case of the single-phase biochar samples, an increase in the temperature for thermal treatment from 400 °C to 850 °C causes a gradual shift in the grain sizes of these samples towards smaller particle sizes.

### 3.2. Thermochemical Conversion and Gasification Process of the Biochar Samples Studied via the Thermal Analysis Method

The key factor in utilising biomass-derived biochar is determining the physicochemical transformations they undergo under the operating conditions of DC-SOFC fuel cells. As mentioned, DC-SOFC fuel cells are started at room temperature and then gradually heated to temperatures of 700–850 °C. During the temperature rise, carbonaceous biomass fuels can change their properties due to the decomposition of mainly organic substances and the release of additional gaseous reagents, which affect the composition of the gas atmosphere in the anode compartment and further chemical reactions between the solid fuel and the anode material. The authors of [39,44,45] pointed out that the process of cellulose pyrolysis and the release of gases, such as H_2_, methane, carbon monoxide (II), methane, and other hydrocarbons, can decisively influence the operating parameters of DC-SOFCs.

**Figure 8:**
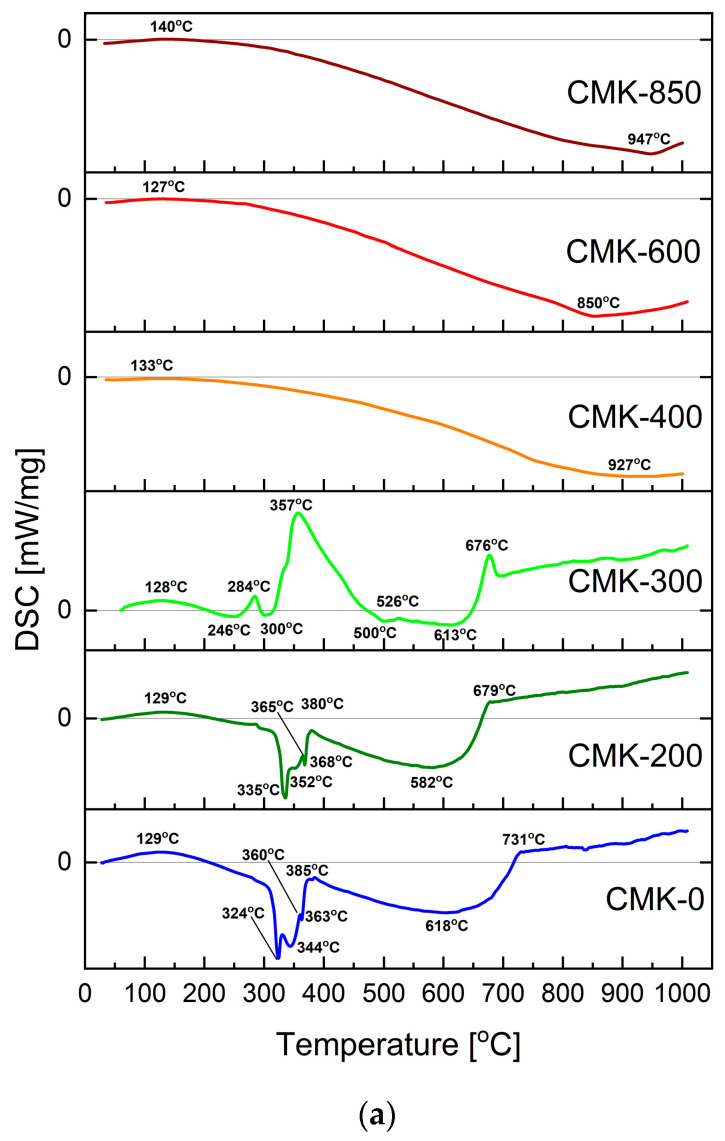

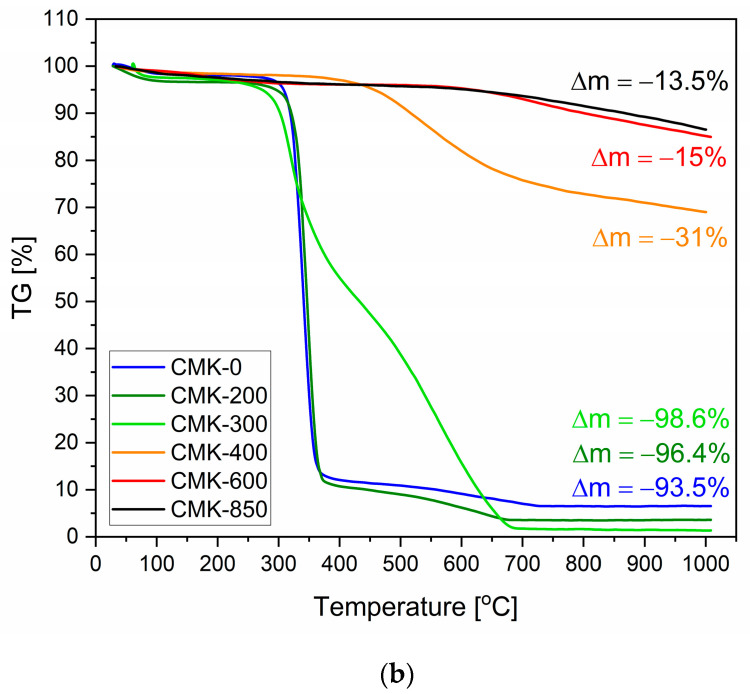
(**a**) Differential scanning calorimetry curves for the raw cellulose CMK-0 and the carbonised samples from the CMK series. (**b**) Thermogravimetric (TG) curves recorded for the raw cellulose CMK-0 and the biocarbon-based samples, from CMK-200 to CMK-850.

Figure 8a shows the differential scanning calorimetry (DSC) curves of the raw cellulose CMK-0 and the carbonised samples from the entire CMK series, from 200 to 850.

Cellulose, one of the main components of plant biomass, undergoes complex chemical reactions during pyrolysis, producing thermodynamically unstable intermediates. The first stage of cellulose pyrolysis is the initial depolymerisation of oligosaccharides. The proposed mechanism is the formation of levoglucosan from the so-called active cellulose [46].

The DSC curve plotted for the raw cellulose sample shows that the gradual heating of the CMK-0 sample from room temperature to ~129 °C led to the onset of cellulose decomposition [47]. Within the 324–360 °C temperature range, some broadened endothermic peaks related to the decomposition of crystalline cellulose could be observed, leading to the formation of organic compounds.

As a result of subsequent reactions, levoglucosan can undergo decomposition, fragmentation, dehydration, decarbonylation, and decarboxylation. During the conversion of levoglucosan at a temperature of about 500 °C, levoglucosenone is formed (directly or via intermediates, such as 1,4:3,6-dianhydro-α-D-glucopyranose or 1,6-anhydro-α-D-glucofuranose). Water, furfural, and 5-hydroxymethylfurfural can dehydrate levoglucosenone. The resulting products can be converted into biochar or light gas products in further steps. Carbon oxides (CO, CO_2_) are formed during decarbonylation or decarboxylation. The yield of the individual intermediate products depends mainly on the temperature of the pyrolysis process and its rate of increase [47,48].

Figure 8b shows the mass losses of the CMK-0 cellulose feedstock sample and the torrefied CMK samples at temperatures from 200 °C to 850 °C.

The TG curve in Figure 8b indicates that the original CMK-0 cellulose sample had a loss of about 93%. The partial torrefaction of the cellulose samples at CMK-200 and CMK-300 led to an increase in mass loss from 96% to approximately 98%. The samples CMK-200 and CMK-300 used for investigations are already partially carbonised compared to the CMK-0 sample. Based on XRD phase composition investigations, it was found that these are two-phase materials composed of a carbon phase with a low degree of order in the crystallographic structure and a residual cellulose matrix. Research carried out using IR spectroscopy methods confirmed the presence in these samples of a large number of functional groups directly related to carbon organic compounds.

Further thermal heating of CMK-200 and CMK-300 samples, which have previously undergone thermal conversion, leads to further thermal decomposition processes with the release of, e.g., oxygen. In turn, the presence of oxygen leads to further conversion processes as a result of further pyrolysis or gasification of biocarbons to the gaseous forms CO and CO_2_. The direct consequence of these phenomena is the formation and release of more and more gaseous products as the temperature increases.

By contrast, for the cellulose samples subjected to pyrolysis at temperatures of 400–850 °C, the observed mass losses were much lower. The loss was ~30% for the CMK-400 sample, while such changes were between ~13% and ~16% for the CMK-600 and CMK-850 samples, respectively.

The solid fuel in the anode chamber of the DC-SOFC cell may also undergo gasification under the influence of steam, oxygen, and CO_2_. Thus, the CMK-850 biochar samples were chosen for reactivity tests with evaporating H_2_O. It should be emphasised that the gasification reaction of carbon particles with H_2_O as a medium may result in the formation of further gaseous products, such as H_2_, CO, and methane (CH_4_), which are valuable fuels for DC-SOFCs.

**Figure 9:**
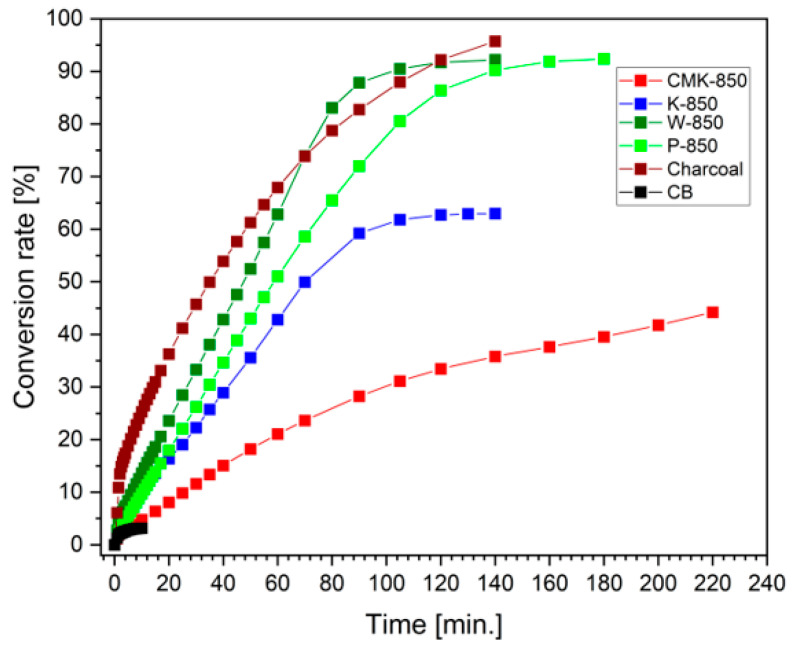
Conversion rate of biochar CMK-850 from the solid-state phase to the gaseous phase during steam gasification. Data from selected chars obtained from wastes under the same experimental conditions is added.

In Figure 9, the conversion rate of biochar sample CMK-850 to the gaseous phase using steam as a medium for gasification is presented. This figure includes data from biochar synthesised at 850 °C from organic waste materials such as coconut shells (K-850), pistachio shells (P-850), walnut shells (W-850), and other carbonaceous materials, including carbon black (CB). Activated charcoal is added for comparison.

Figure 9 illustrates that the CMK-850 sample was characterised by a relatively low degree of conversion from the solid phase to the gas phase. Therefore, a much higher degree of carbon conversion in an atmosphere of H_2_O vapour is characteristic of samples of chars obtained from waste agrifood products. The comparative results indicated the impact of such features as mineral inorganic compounds (e.g., alkaline oxides and Fe_2_O_3_), which act as catalysts for gasification in an H_2_O atmosphere.

**Figure 10:**
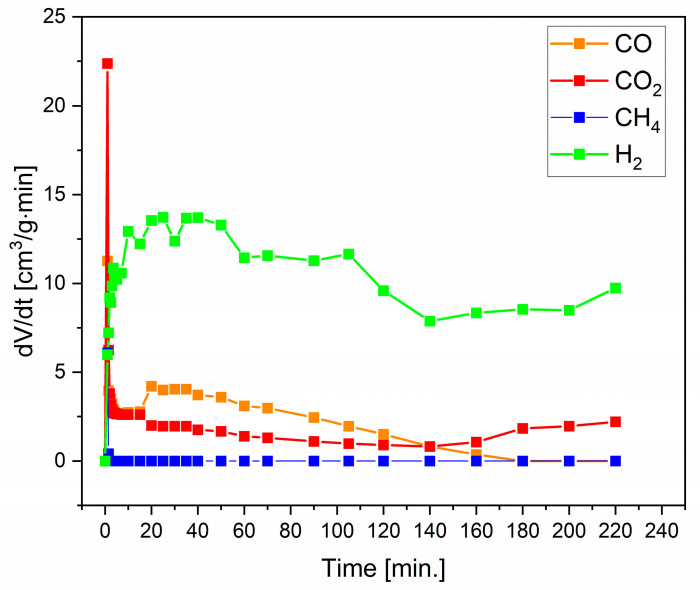
Curves of dV/dt versus time recorded for generated gases, such as CO, CH4, H2, and CO2, during the steam gasification of biochar CMK-850 at 850 °C.

Based on the dependencies of dV/dt versus time (Figure 10) recorded for the produced gases, such as CH_4_, CO, H_2,_ and CO_2_, the highest reaction rate of H_2_ gas formation for dV/dt was recorded from the beginning of the process until about 45 min. Further exposure to H_2_O vapour led to a decrease in the amount of H_2_ produced. By contrast, CH_4_ formation could only be observed in the initial phase of the reaction (up to 5 min), after which it significantly decreased. The next group of gas products to be analysed were CO and CO_2._ The most significant increase in the volume of CO and CO_2_ was observed in the first 10 min of the reaction.

**Figure 11:**
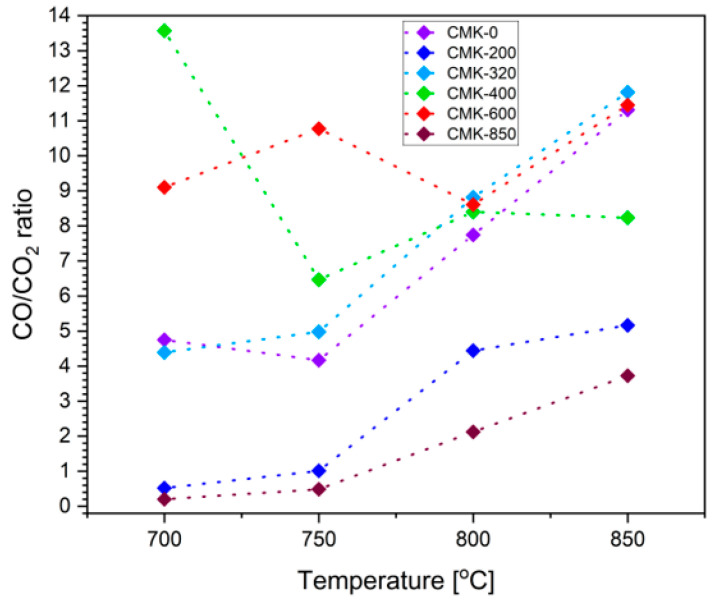
Dependence of changes in the CO/CO_2_ ratio determined in the anode space of the direct carbon solid oxide fuel cell.

A crucial issue for explaining the chemical and electrochemical processes that took place in the deposit was the analysis of the ratio of the gas products (i.e., CO/CO_2_ formed in the solid fuel bed), which resulted from placing a powdered biochar sample in the anode space of the tested DC-SOFC. Figure 11 shows the dependence of the ratio variation in CO/CO_2_ gaseous products at the outlet of the anode space of the DC-SOFC on temperature. Data were recorded for all biocarbon-based samples obtained from cellulose feedstock. The temperatures indicated refer to the biochar bed placed in the anode space; they varied between 700 °C and 850 °C.

According to Figure 11, the ratio of CO/CO2 gaseous products released from the anode space of the SOFC for all tested CMK series biofuels increased with the operating temperature of the DC-SOFC. The primary explanation for this phenomenon is that as the temperature rises, the formation of carbon monoxide (II) due to the reaction of carbon and CO with temperature is significant. Hence, the highest CO/CO2 ratio in the entire temperature range was characteristic of the CMK-0 sample, and the CMK-200 and CMK-320 samples were thermally treated, respectively, at 200 °C and 320 °C. However, slightly different changes in the CO/CO_2_ gas product ratio were recorded for the CMK-400 and CMK-600 samples. The lowest index values were noted in the CMK-850 sample. Furthermore, based on the XRD and Raman spectroscopy investigations, it was found that among the single-phase biochar samples, the CMK-400 sample is characterised by the highest degree of disordered carbon crystallographic structure as well as by the presence of a large number of adsorbed functional groups typical of organic carbon compounds. Additional thermal heating of the CMK-400 sample and the CMK-600 sample in the form of a small carbon bed in the anode chamber leads to further thermal conversion associated with the decomposition of organic compounds. This phenomenon is accompanied by the release of larger amounts of CO and CO_2_ as decomposition products, depending on the temperature of the fixed carbon bed. Under these conditions, the gas atmosphere surrounding the biocarbon bed is more complex, and chemical compositions can be dynamically changed. This phenomenon has a direct effect on the changes in the CO/CO_2_ ratio in the temperature range of 700–800 °C.

At higher temperatures, the Boudouard reaction between solid carbon and surrounding CO_2,_ which leads to the formation of CO, can have a greater impact than at lower temperatures. Similar phenomena can take place when the CMK-600 is placed in the anode chamber of the DC-SOFC. In the case of the CMK-600 sample, the dynamic changes in the ratio CO/CO_2_ at the temperatures (700–800 °C) are lower compared to the CMK-400 sample.

An important issue regarding the development of a strategy for selecting an organic waste raw material is the problem of the impact of Boudouard reaction catalysts on the kinetics of the CO formation process, as they will undergo electrochemical oxidation at the anode during DC-SOFC operation. According to reactions C + O^2−^→CO_2_ + 4e^−^ and C + O^2−^→CO + 2e^−^, the product of the electrochemical oxidation will be gaseous CO_2_.

**Figure 12:**
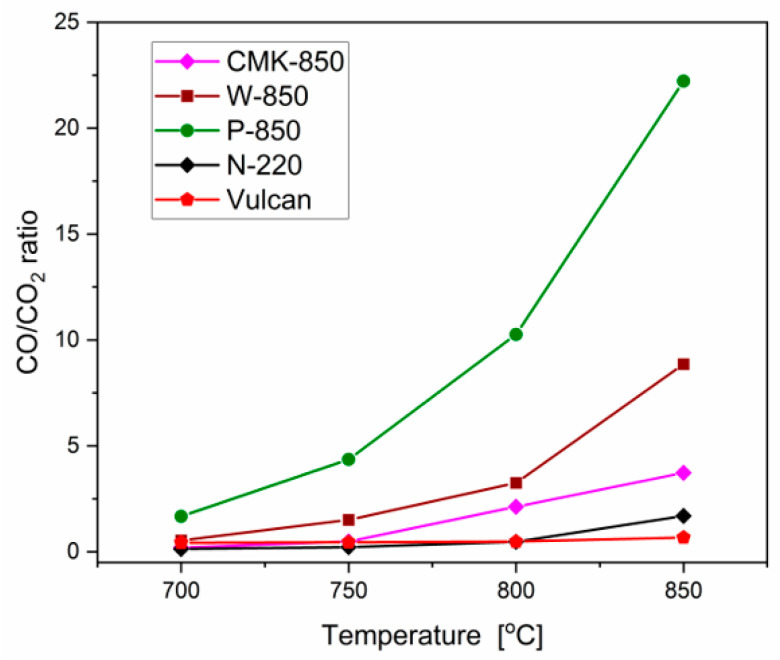
CO/CO_2_ ratio versus temperature of the carbon bed in the anode chamber of the direct carbon solid oxide fuel cell for various carbonaceous materials.

Figure 12 shows a comparison of changes in the CO/CO ratio based on chromatographic measurements of these gas products. CO and CO_2_ gas products were created in the anode space, where the solid fuel bed was placed. The DC-SOFC cell did not operate under these conditions.

Based on the comparison in Figure 12, the highest CO/CO_2_ ratios were obtained for the biochar samples from pistachio and walnut shells. The CO/CO_2_ values confirmed the key role of mineral residues in the chemical processes that occurred in the solid fuel deposit, including the reverse Boudouard reaction. Another important feature was the high surface area of the samples. The surface area of the biochars obtained from walnut shells, pistachio shells, and activated carbon varied between ~300 m^2^/g and 1000 m^2^/g.

The biochar sample obtained from CMK-850 had a lower proportion of CO/CO_2_ gas products compared to biochars obtained from biomass waste. The lowest CO/CO_2_ values were observed in synthetic carbon black CB. Thus, it appears that more CO as an important product of gasification can be expected in the case of carbon materials having built-in alkali oxides in their structure or other metals that have good catalytic properties for the Boudouard reaction [48,49,50,51].

### 3.3. Investigation of the Biocarbon Oxidation Process Studied on the Surface of 8 mol% Y_2_O_3_ in ZrO_2_ (8YSZ) Oxide Electrolyte

In [52,53,54], the authors showed that the electrochemical oxidation of carbon fuel according to reaction: C + O_2_ → CO_2_ takes place on the surface of the yttria-stabilised zirconium oxide (YSZ) electrolyte. Nürnberger et al. [55] investigated the performance of DC-SOFCs using synthetic carbon-based materials by placing a dry-pressed carbon pellet (Vulcan XC-72 carbon black or graphite) directly on the 3YSZ surface. Electrochemical measurements of the DC-SOFCs were carried out using nitrogen as a purge gas in the anode chamber. The researchers noticed that the physicochemical properties of the synthetic carbon materials used as fuels have a decisive effect on the parameters of DC-SOFCs, such as open circuit voltage, current density, and power density. They also showed that non-crystalline (amorphous) CB fuels perform better than graphite-based materials.

All experimental results and subsequent calculations confirmed that the reverse Boudouard reaction for CO generation plays a less significant role in the overall performance of DC-SOFCs. Carbon-based synthetic materials constitute one group of solid fuel cells for DC-SOFCs [56,57].

The second group of eligible solid fuels for DC-SOFCs are readily available biocarbon-based fuels derived from non-woody or woody biomasses. The electrochemical oxidation process is more complicated for biocarbon particles derived from cellulose or organic waste due to additional chemical compounds as residual components of the starting material’s thermal conversion process. The adsorbed pyrolytic gases containing hydrocarbons, alcohols, and other organic compounds in the biochar bed can alter the pathways of the electrochemical reactions and significantly impact DC-SOFC performance [58,59].

Thus far, this phenomenon has not been investigated in samples of high-purity organic materials. As the MIR and XPS spectroscopy results showed, various functional groups were adsorbed on the surface of the powdered carbon particles, indicating the presence of other substances that could decompose further at higher temperatures and serve as gaseous or solid fuel for the electrochemical oxidation of the anode.

**Figure 13:**
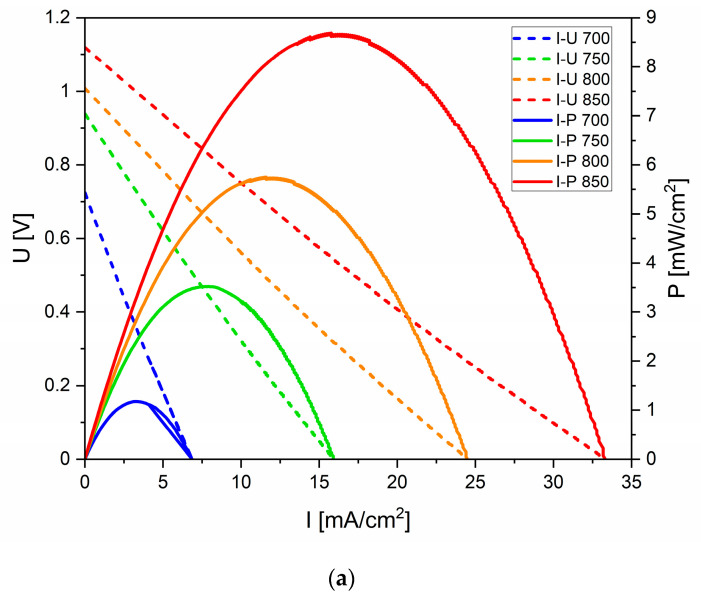

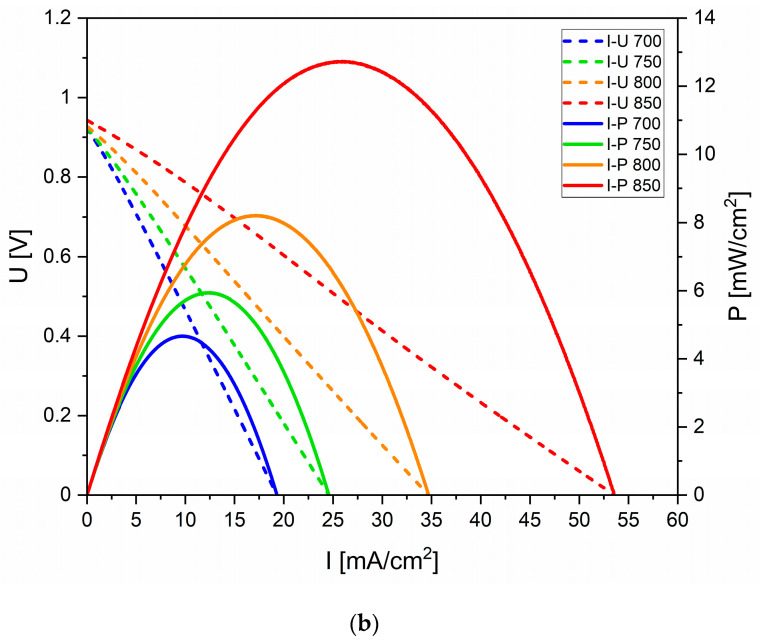
(**a**) Dependence of voltage–current density (U–I) and power–current density (P–U) of DC-SOFC (A) supplied by cellulose sample CMK-0 as a solid fuel. (**b**) Dependence of voltage–current density (U–I) and power–current density (P–I) of DC-SOFC (A) supplied by cellulose sample CMK-850 as a solid fuel.

The analysis of I (mA/cm^2^), as well as electrical P obtained from the investigated DC-SOFC (A), determined that within the temperature range of 700–850 °C, P slightly increased with a higher temperature but was still rather low. Figure 13b presents the voltage (U), I and P dependence curves of the DC-SOFC (A) supplied by the CMK-850 biochar.

Based on the U–I and P–I curves in Figure 13a,b for the DC-SOFC (A) supplied by the CMK-0 and CMK-850 biochar samples, respectively, it could be concluded that an increase in the DC-SOFC’s operating temperature from 700 °C to 850 °C led to an increase in I and P. The DC-SOFC was turned on at a room temperature of 20 °C and then warmed up to an operating temperature of 700–850 °C. Simultaneously, the cellulose sample from CMK-0 in the anode chamber of the DC-SOFC underwent gradual conversion to biochar.

**Figure 14:**
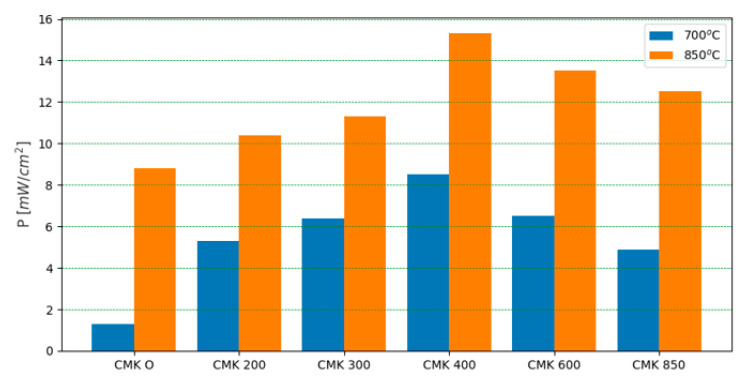
Effect of biochar pyrolysis temperature on the variation in electrical power (P) of DC-SOFC (A). Data are recorded for 700 °C and 850 °C.

The CMK-850 sample was a biocarbon that was pyrolyzed at the highest temperature of 850 °C. As in the case of the DC-SOFC supplied by the CMK-850 biochar powder, the values of I and P were higher than for the DC-SOFC supplied by the CMK-0 cellulose. The effect of the temperature of the biochar pyrolysis on DC-SOFC (A) performance is presented in Figure 14.

Looking at the variations in P for the DC-SOFC fuel cells, the DCSOFC (A) supplied by the raw cellulose CMK-0 exhibited the lowest electrochemical performance. The main reason for the carbon particles’ very low electrochemical activity for oxidation into CO_2_ on the 8YSZ surface was the lowest carbon content (~42 wt%) in the original cellulose powder used to supply the DC-SOFC. Another reason was that in the first stage, no conductive cellulose particles had direct contact with oxide ions (O^2−^) conducting the 8YSZ electrolyte. The carbon particles were formed in situ inside the anode chamber when the DC-SOFC was initially heated.

In turn, the partial carbonisation of the cellulose samples and the formation of torrefied solid fuel (CMK-200 or CMK-300) led to an increase in DC-SOFC (A) performance. The highest values were obtained for the DC-SOFC supplied by the CMK-400 sample. The partial increase in the temperature of cellulose carbonisation above 600 °C and the subsequent use as a solid fuel to supply the DC-SOFCs could lead to a slight decrease in the P of the DC-SOFCs. There are a few possible explanations for this phenomenon.

The rise in temperature during biocarbon synthesis led to an increase in the graphitization of the carbon particles in the biochars. The increase in particle size of biochar CMK-850 may have also led to a decrease in the DC-SOFC’s electrical performance. Previous papers [32,60] postulated that solid fuels with a non-crystalline carbon particle structure, high surface area, and low particle size of carbon powder can extend the electrochemical reaction zone, which contributes to the direct contact of carbon particles with oxide electrolyte.

Furthermore, a possible reason for the difference in the P of the investigated DC-SOFC (A) was the electrochemical oxidation of adsorbed biochar bed different hydrocarbons or organic compounds that were formed during cellulose decomposition. It is well known that dry hydrocarbons, such as CH_4_ and propane, can be oxidised in DC-SOFCs. The increase in temperature of DC-SOFC (A) above 800 °C caused the thermal decomposition of dry hydrocarbons into H_2_ and carbon [61,62,63,64,65].

**Figure 15:**
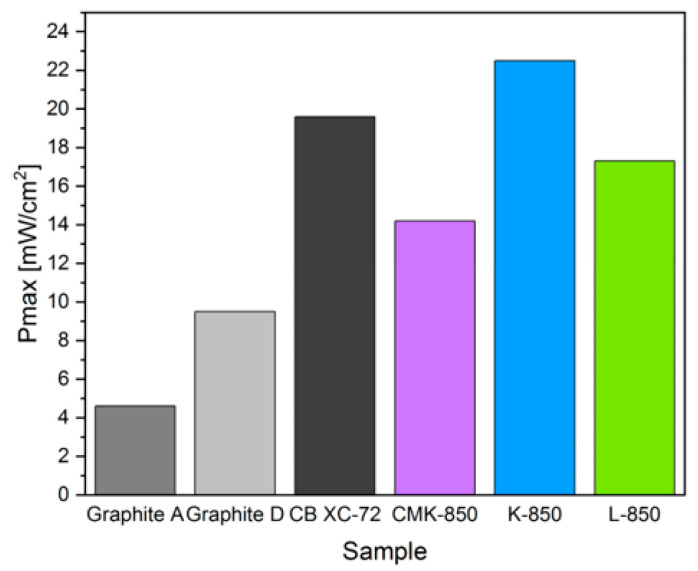
Electrical power output Pmax (mW/cm^2^) obtained from DC-SOFC (B) supplied by the CMK-850 sample and different biochars obtained from waste organic materials, as well as graphite or carbon black used as reference solid fuels. The data refer to a temperature of 850 °C.

Figure 15 is a direct comparison of the Pmax (mW/cm^2^) of the DC-SOFC (A) supplied by CMK-850 and different biochars obtained from waste organic materials, as well as graphite and CB used as reference solid fuels.

As seen in Figure 15, the DC-SOFC powered by biochar from coconut shells showed higher P values compared to the DC-SOFC supplied by CMK-850 biochar. The DC-SOFC powered by biochar obtained from hazelnut shells and low-crystallinity carbon XC-72 also exhibited better Pmax compared to the DC-SOFC supplied by biochar obtained only from cellulose. A direct comparison of these values showed that the CMK-850 biochar sample obtained from cellulose had lower electrochemical reactivity in the anodic electrochemical oxidation of carbon particles, according to reaction C + O^2−^→CO_2_ + 4e^−^, compared to the other biocarbons from waste shells.

**Figure 16:**
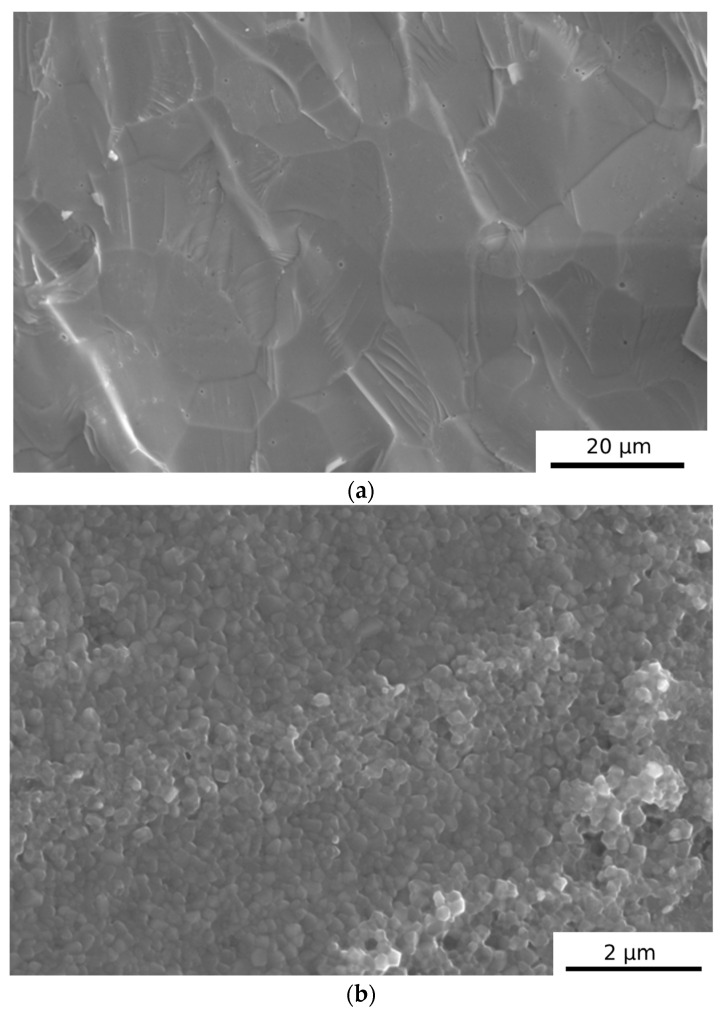
(**a**) Microstructure of 8YSZ electrolyte (A1) sample sintered at 1600 °C for 2 h. (**b**) Microstructure of 8YSZ electrolyte (A2) sample sintered at 1230 °C for 2 h.

Thus far, the influence of electrolyte surface development (i.e., the selection of fine-crystalline or nanocrystalline electrolytes) during the electrochemical oxidation of carbon has not been analysed. Figure 16a,b show the microstructures of 8YSZ disc electrolytes with different microstructures. The 8YSZ electrolyte sample sintered at 1500 °C for 2 h was characterised by an average grain size of 3–5 μm, while the 8YSZ sample sintered at 1230 °C for 2 h was characterised by an average grain size of 0.2–0.4 μm.

These experiments illustrated that an electrolyte with a fine-grained structure could obtain a slightly higher P from DC-SOFC (A1). The electrical ionic conductivity of the microcrystalline sample 8YSZ (A1) was higher than that of the polycrystalline sample 8YSZ (A2). The energy activation of the 8YSZ sample (A1) was lower than that of the 8YSZ sample (A2). Table 3 shows electrical conductivity (S/cm) and energy activation in the temperature range of 500–850 °C. The data on the performance of the DC-SOFC with electrolytes (A1) and (A2) are included.

Table 3 confirms that the factor limiting high I and P obtained from DC-SOFC was the electrochemical carbon oxidation reaction to carbon dioxide (reaction C + O^2−^→CO_2_ + 4e^−^), which occurred only on the surface of the 8YSZ electrolyte.

### 3.4. Electrochemical Performance of DC-SOFC (B) Supplied by Solid Fuels Obtained as Biochar from Cellulose Feedstock

**Figure 17:**
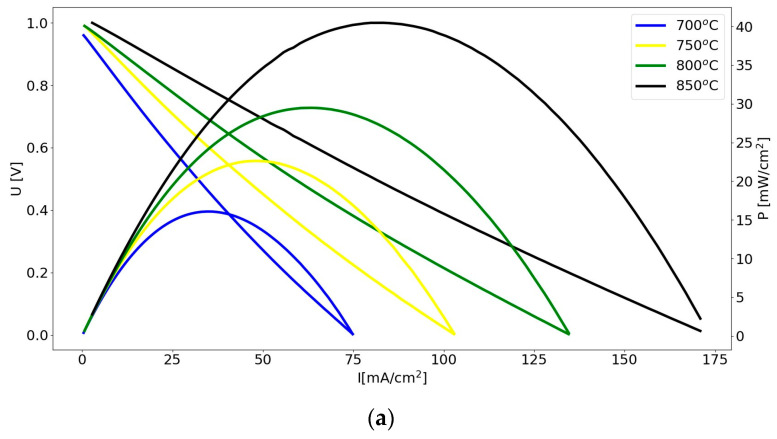

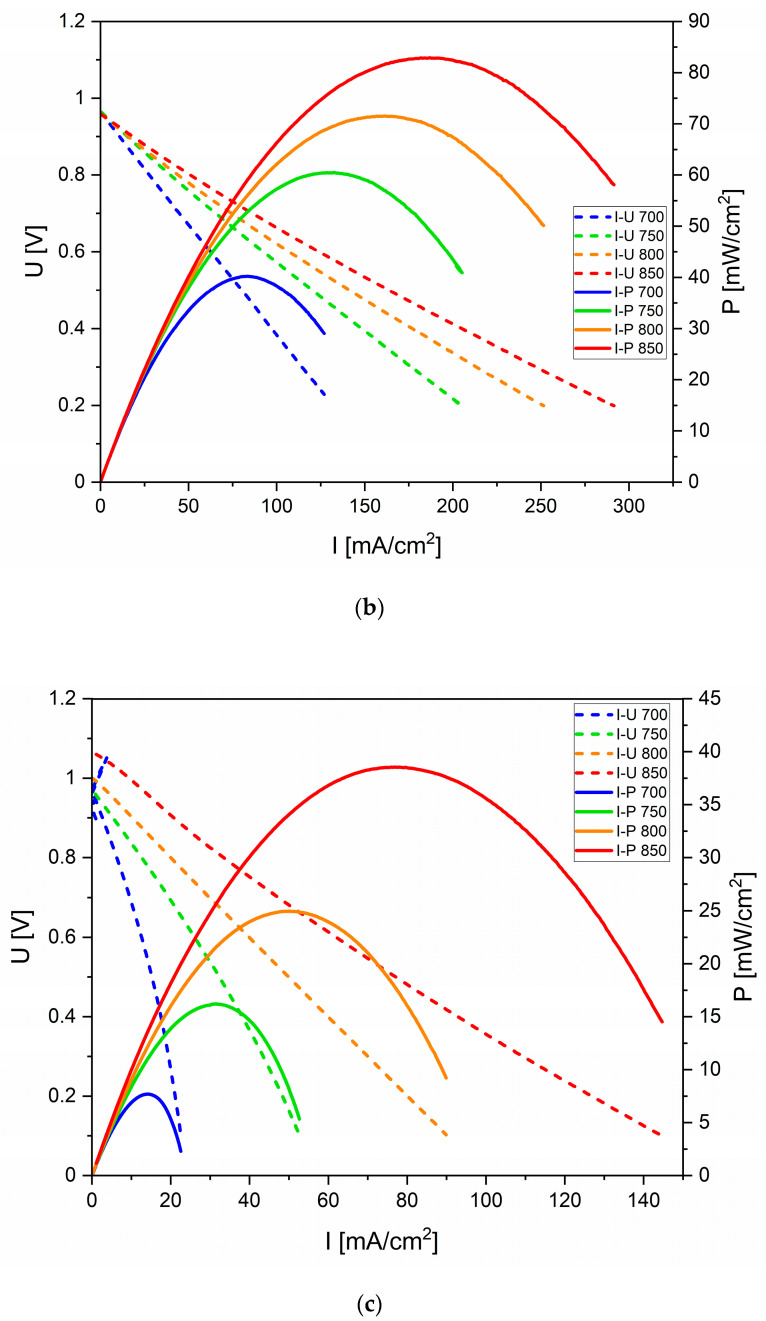
(**a**) Voltage–current density (U–I) and power–current density (P–I) curves of the DC-SOFC (A) supplied by CMK-0 fuel. (**b**) Voltage–current density (U–I) and power–current density (P–I) dependencies of the DC-SOFC (B) supplied by CMK-400. (**c**) Dependencies of voltage–current density (U–I) and power–current density (P–I) of the DC-SOFC supplied by the CMK-850 biochar.

Electrochemical investigations of DC-SOFC (B) performance were conducted to gain new knowledge of how the physiochemical properties of biocarbon samples from CMK-0 to CMK-850 influence the performance of DC-SOFCs with Ni-YSZ cermet anodes. The U–I and P–I curves of the DC-SOFC (A) supplied by the initial CMK-0 microcrystalline cellulose powder are presented in Figure 17a.

In the case of DC-SOFCs with Ni-based cermet anodes, the electrochemical oxidation of solid carbon particles has a more complicated mechanism than described by the equation C + O^2−^ → CO_2_ + 4e^−^ or a combination of two equations, C + O^2−^ → CO + 2e^−^ and CO + O^2−^ → CO_2_ + 2e^−^.

If the raw cellulose CMK-0 sample is used as a solid fuel in a DC-SOFC, the mechanism of electrochemical oxidation of solid fuels in DC-SOFC (B) most responsible is the pyrolytic mechanism [64]. It should be emphasised that when the fuel cell is heated from ambient temperature to temperatures of 700–850 °C, in situ decomposition of organic substances occurs with the release of large amounts of gaseous products.

In such conditions, some gasification of carbon particles to CO or CO_2_ due to the presence of residual oxygen on the biocarbon surface can occur. Thermal analysis and DTA-TG tests revealed that the mass loss was approximately 83% for raw cellulose, and the resulting gaseous, liquid, and solid products may have had a significant impact on the electrochemical pathways of the oxidation fuels on the Ni-YSZ surface.

All pyrolytic gases that are products of cellulose decomposition, such as H_2_, CO, and CxHy, can easily be transported to an anode, where they can be electrochemically oxidised. The gaseous organic compounds can also be adsorbed on the surface of the anode and the pores occurring in all volumes of the Ni-YSZ cermet anode. During the heating of DC-SOFC from 20° to 850 °C, various thermal decomposition processes occur in the cellulose matrix as well as on the biocarbon bed.

Analysis of the DSC-TG tests showed that a small amount of solid carbon fuel was obtained from cellulose decomposition. Only 13 wt% of biochar in solid form could be formed in the anode chamber. Moreover, the Pmax of DC-SOFC (B) was limited to 10–40 mW/cm.

Figure 17b shows the U–I and P–I dependencies of the DC-SOFC (B) fed with the CMK-400 sample.

Figure 17c shows the U–I and P–I dependencies of the DC-SOFC supplied by the CMK-850 biochar sample.

Comparing Figure 17b,c shows that lower I and P were attained from the DC-SOFC (B) supplied by biochar carbonised at 850 °C compared to the biocarbon fuels obtained in the temperature range of 200 °C to 850 °C. One of the reasons for the deterioration of the operating parameters of the cell may have been the increase in the degree of graphitization of carbon particles in the CMK-850 biochar in relation to the biochar samples synthesised at a lower temperature.

Another reason may have been that the CMK-850 sample was characterised by the lowest mass loss; therefore, the share of the gaseous part in the anodic process of electrochemical oxidation should also be considerably lower. During the SEM observation, the biochar sample synthesised at 850 °C had very few pores in the carbon particles. This lack of porosity could have caused difficulties in CO delivery to the Ni-YSZ anode. Antunes et al.’s [65] numerical model and experimental results illustrated that the reaction mechanism of the electrochemical oxidation of char obtained from lignite is limited by the electro-oxidation of CO in the anode, and the gas transport I is driven mainly by natural convection from the small carbon bed formed in the anode chamber to the surface of the anode.

**Figure 18:**
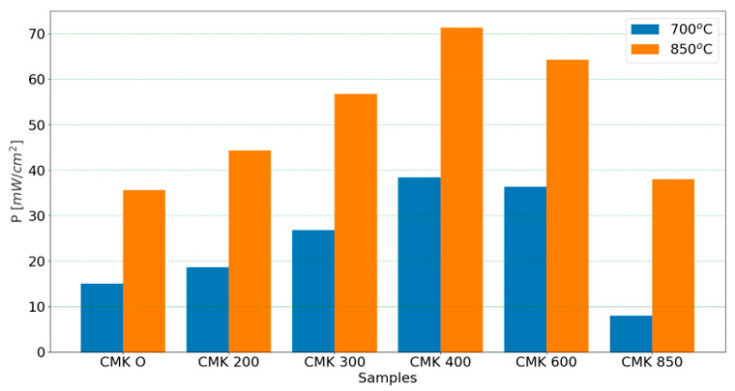
Variation in the electrical power output of DC-SOFC (B) supplied by different solid fuels.

Biochar samples obtained from microcrystalline cellulose are characterised by a lack of mineral substances but have a high content of gaseous products originating from the decomposition of individual cellulose fractions. Figure 18 presents the electrochemical P at 700 °C and 850 °C of a DC-SOFC (B) powered by solid fuels from the CMK series. The highest P was obtained for the DC-SOFC supplied by the CMK-400 biochar. Increasing the thermal treatment temperature of the biochar from 600 °C to 850 °C led to a decrease in the P of the DC-SOFC (B).

The best operating parameters of the DC-SOFCs were obtained from the partially developed biochar samples, which were characterised by a relatively high carbon content and a low degree of graphitization. The CMK-320 and CMK-400 biochar samples exhibited large mass losses. Moreover, the conditions for biochar synthesis corresponded to when most or complete cellulose decomposition occurred. The CMK-320 and CMK-400 conditions were characterised by the highest degree of disorder. Increasing the carbonisation temperature led to an increase in the degree of order, and further thermal processes and biochar formation may have occurred during the heating of the DC-SOFC.

**Figure 19:**
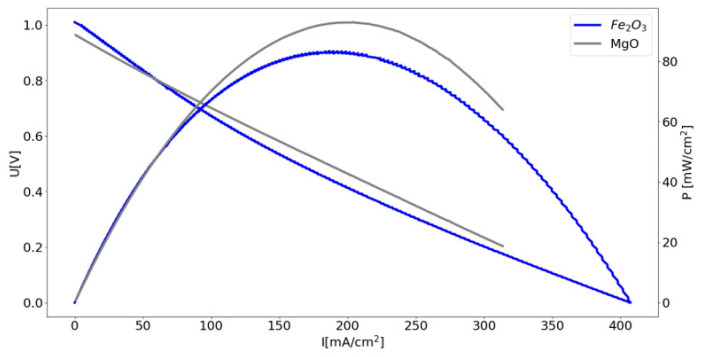
Dependence of voltage–current density (U–I) and power–current density (P–I) of DC-SOFC (A) supplied by cellulose sample CMK-850 +5% wt. Fe_2_O_3_ or CMK-600 +5% wt. MgO. As seen in Figure 19, adding a Fe_2_O_3_ or MgO catalyst to the biochar increased the operating parameters of the DC-SOFC compared to the DC-SOFC powered by carbon fuel only without the catalyst (Figure 17c). The improved biochar activity in the reverse Boudouard reaction was necessary to enhance the electrical P of the DC-SOFC (B) [67].

In the case of the DC-SOFCs supplied by biochars synthesised within the temperature range of 400–850 °C, Gur’s [65,66] proposed ‘CO shuttle mechanism’ may have also played a role in the reaction pathways of the DC-SOFC (B). The main feature of the shuttle mechanism is the delivery of the oxidant CO_2_ as a product of electrochemical reactions to solid carbon fuel via a chemical vehicle. CO, which is formed in the bed and then oxidised at the anode to form CO_2_, interacts with the carbon bed to form more CO. Another way to increase the operating parameters of a DC-SOFC (B) may be to add a Boudouard reaction catalyst to the fuel. Figure 19 shows the U–I and P–I dependencies of a DC-SOFC (B) powered by CMK-850 + 5% wt Fe_2_O_3_ or CMK-850 + 5% wt MgO. The data refer to a temperature of 850 °C.

## 4. Conclusions

This paper presents the results of research into the physicochemical properties of biocarbon samples obtained from cellulose feedstock. These samples were subjected to torrefaction and carbonisation processes in the temperature range of 200–850 °C in an inert gas atmosphere. As such, biochar samples obtained from cellulose were used as solid carbon fuels for supplying DC-SOFC. The analysis of XRD patterns recorded for all the biocarbon-based samples from cellulose showed that monophase biocarbon was attained at temperatures above 300 °C. A chemical elemental analysis showed that thermal treatment ranging from 200 °C to 850 °C led to the synthesis of biocarbon powders with a carbon content of 40% to 90%. The biochar sourced from cellulose feedstock had a low degree of graphitization. The particle size distribution and morphology of the biocarbon samples from cellulose revealed that they mostly possessed isometric particle shapes and a small amount of elongated, needle-like shapes.

Using this series of biochar samples in the study of solid fuel gasification and electrochemical processes allowed for the acquisition of new knowledge regarding the design of solid fuels to power DC-SOFCs. Studies of the gasification processes taking place in the H_2_O atmosphere have shown that char obtained from cellulose has lower activity in relation to biochar sourced from organic waste materials from agri-food production. The lower chemical activity of fully crystallised biochar obtained from cellulose was compared to biochar obtained from pistachio and walnut shells. The main difference is the lack of mineral substances (mainly alkali oxides and iron), which play a key role in gasification processes. On the other hand, biochar samples from cellulose were characterised by greater activity in the gasification processes compared to high-purity synthetic carbons (e.g., graphite and carbon black powders).

An examination of the electrochemical oxidation process of the obtained biochar revealed that the temperature of biochar synthesis and its physicochemical properties had a significant impact on the electrochemical oxidation of carbon particles on the surface of the oxide-conducting electrolyte. This study found that the application of a fully disordered biochar sample, which was synthesised at 400 °C, obtained the highest electrical performance of DC-SOFC. This sample consisted mostly of disordered and isometric carbon particles. The increase in temperature of biochar synthesis up to 600 °C led to a small deterioration in DC-SOFC performance. The lowest values of current density (I) and power density (P) were obtained for the biochar sample synthesised at a higher temperature of 850 °C. The main reason for such behaviour was the increased degree of graphitization of the samples. The analysis found that the impact of surface and microstructure of 8%mol Y_2_O_3_ in ZrO_2_ (8YSZ) solid electrolytes was lower compared to the properties of carbon-based fuels. Comparing the trend of changes in the electric power P of the DC-SOFC involving the Ni-8YSZ anode, it was found that the highest values of power output, Pmax, were obtained for the biochar samples synthesised at 400 °C. A further increase in temperature led to a decrease in DC-SOFC performance. One of the reasons for this was the increase in graphitization of carbon particles. The second was the low concentration of CO in the anode chamber. The catalysts for the reverse Boudouard reaction occurring in a biocarbon bed were critical to ensuring high performance and stable operation under electrical load, which is crucial for DC-SOFC development.

## Figures and Tables

**Table 1:** Nomenclature of the biocarbon series samples obtained from cellulose.

Sample Name	Pyrolysis Temperature [°C]	Sample Name	Pyrolysis Temperature [°C]
CMK-0	None	CMK-320	320
CMK-200	200	CMK-400	400
CMK-250	250	CMK-600	600
CMK-300	300	CMK-850	850

**Table 2:** Data obtained for elemental analysis.

Sample	C	H	S
CKM-0	41.6	9.77	0.00
CKM-200	42.1	9.30	0.00
CKM-250	51.4	8.74	0.00
CKM-300	55.7	4.73	0.00
CKM-320	62.0	4.06	0.00
CKM-400	75.5	3.34	0.00
CKM-600	83.9	1.83	0.00
CKM-850	90.1	1.43	0.01

**Table 3:** Characteristic features of DC-SOFCs (A) constructed from 8YSZ electrolyte (A1) and 8YSZ electrolyte (A2).

Object	Parameter	Value
8YSZ sample (A1)	Total electrical conductivity σ (S/cm) at 850 °C	4.1 × 10^−2^ (S/cm)
	Energy activation (eV) in the temperature range of 500–850 °C	0.91 eV
8YSZ sample (A2)	Total electrical conductivity σ (S/cm) at 850 °C	8.6 × 10^−2^ (S/cm)
	eV in the temperature range of 500–850 °C	0.85 eV
DC-SOFC (A1)	Electrical power output of CMK-400 fuel at 850 °C	14.3 mW/cm^2^
DC-SOFC (A2)		21.6 mW/cm^2^
DC-SOFC (A1)	Electrical power output of CMK-850 fuel at 850 °C	12.1 mW/cm^2^
DC-SOFC (A2)		16.8 mW/cm^2^

## Data Availability

The original contributions presented in the study are included in the article, further inquiries can be directed to the corresponding authors.
